# Inner Ear Disease and Benign Paroxysmal Positional Vertigo: A Critical Review of Incidence, Clinical Characteristics, and Management

**DOI:** 10.1155/2011/709469

**Published:** 2011-08-02

**Authors:** M. Riga, A. Bibas, J. Xenellis, S. Korres

**Affiliations:** ^1^ENT Department, University Hospital of Alexandroupolis, Democritus University of Thrace, Dragana, 68100 Alexandroupolis, Greece; ^2^ENT Department, Hippokrateion General Hospital of Athens and National University of Athens, 11527 Athens, Greece

## Abstract

*Background*. This study is a review of the incidence, clinical characteristics, and management of secondary BPPV. The different subtypes of secondary BPPV are compared to each other, as well as idiopathic BPPV. Furthermore, the study highlights the coexistence of BPPV with other inner ear pathologies. *Methods*. A comprehensive search for articles including in the abstract information on incidence, clinical characteristics, and management of secondary BPPV was conducted within the PubMed library. *Results*. Different referral patterns, different diagnostic criteria used for inner ear diseases, and different patient populations have led to greatly variable incidence results. The differences regarding clinical characteristics and treatment outcomes may support the hypothesis that idiopathic BPPV and the various subtypes of secondary BPPV do not share the exact same pathophysiological mechanisms. *Conclusions*. Secondary BPPV is often under-diagnosed, because dizziness may be atypical and attributed to the primary inner ear pathology. Reversely, a limited number of BPPV patients may not be subjected to a full examination and characterized as idiopathic, while other inner ear diseases are underdiagnosed. A higher suspicion index for the coexistence of BPPV with other inner ear pathologies, may lead to a more integrated diagnosis and consequently to a more efficient treatment of these patients.

## 1. Introduction

Benign paroxysmal positional vertigo (BPPV) is the most common vestibular disorder in adults, with a lifetime prevalence of 2.4% [1]. Clinical and laboratory researches have revealed that BPPV is caused by vestibular lithiasis. Dense particles, most likely displaced otoliths, provoke an abnormal deflection to the cupula (a) most commonly when free-floating in the semicircular canals (SCCs) (canalithiasis), (b) when attached to, or impinging upon, a cupula (cupulolithiasis), or (c) most rarely when jammed in a canal or cupula (canalith jam). In any of these conditions, the abnormal deflection of the cupula induces vertigo, that can be severe and incapacitating, as well as nystagmus in the plane of the involved SCC [2]. The mechanism of detachment of the otoconia is not fully understood. It seems that any inner ear disease that detaches otoconia and yet does not totally destroy SCC function can induce secondary BPPV. The most commonly recognised conditions associated with secondary BPPV are head trauma, vestibular neuritis, Ménière's disease, and postsurgical. Other lesions that affect the inner ear and have been implicated in the pathogenesis of secondary BPPV are sudden sensorineural hearing loss and migraine. Ideally, for a causative association to be strong, BPPV should be ipsilateral to the associated condition and symptoms should develop at the same time or after the development of the primary condition. In some cases, it is not clear whether there exists a true causative effect or there is a coincidental association. In most patients with BPPV, a direct association with an ipsilateral disease process affecting the labyrinth can not be identified and idiopathic BPPV remains the most common diagnosis.

To date, few studies have focused on secondary BPPV, which may often be an underdiagnosed entity. The aim of this study was to review the incidence, clinical characteristics, and management of secondary BPPV. Despite the obvious similarities, differences between the clinical manifestations and the outcome of repositioning manoeuvres between the several types of secondary and idiopathic BPPV seem to dictate different diagnostic, counselling, treatment, and follow-up strategies. Another target of this study is to highlight the coexistence of BPPV with a number of pathologies that are also typically associated with dizziness. In these cases, BPPV is often underdiagnosed, because dizziness is attributed to the primary pathology. A number of BPPV patients may describe the resulting vertigo in a rather atypical way, while further testing might reveal typical BPPV [3, 4]. Although less frequently, the reverse may also be true. A significant number of BPPV patients may not be subjected to a full audiological and neurotological examinations and characterised as idiopathic, while other inner ear diseases are underdiagnosed. A higher suspicion index for the coexistence of BPPV with other inner ear pathologies, together with the appropriate examinations, may lead to a more integrated diagnosis and consequently to a more efficient treatment.

## 2. Methods

A comprehensive search for articles regarding BPPV was conducted within the PubMed library attributed 599 articles (search string: {benign paroxysmal positional vertigo OR positional vertigo OR benign paroxysmal vertigo} with Limits used in Title and English). Studies that did not include in the abstract information on incidence, clinical characteristics, and management of secondary BPPV were excluded from the study. A manual cross-reference search of the bibliographies of included papers was carried out to identify additional potentially relevant studies.

## 3. Results

Based on the inclusion in the abstract information on incidence, clinical characteristics, and management of secondary BPPV, 33 papers were initially included in this review. The manual cross-reference search of the bibliographies of included papers substantially increased the number of reviewed papers into 55. For reporting incidence, only large studies of >100 patients were included in this review [5–13]. Variation in the studies' methodology has in some cases complicated comparisons. For example, some studies do not state whether the inner ear disease predated BPPV or the coexisting pathology was only present and on which side. In some studies, head trauma is considered as an event in terms of idiopathic BPPV and therefore not a cause of secondary BPPV [9]. The prognostic role of the simultaneous diagnosis of BPPV and vestibular neuritis, idiopathic sensorineural hearing loss, or Ménière's disease has not been adequately addressed in the literature in terms of both vestibular rehabilitation and recovery from the primary inner ear disease. The same seems to apply for the therapeutical outcome of repositioning manoeuvres in postsurgical BPPV patients.

## 4. Discussion

### 4.1. Incidence and Possible Pathogenetic Mechanisms

A wide variation of incidence of secondary BPPV (3–66%) is observed across studies [5–13]. It is of note that in two large studies by Karlberg et al. (2847 subjects) [9] and Caldas et al. (1271 subjects) [11], the incidence of secondary BPPV varied considerably between 3% and 25.2%, respectively. This may reflect different referral patterns, different diagnostic criteria used for inner ear diseases, and different patient population. The commonest pathologies underlying the induction of secondary BPPV (as percentage of all cases of BPPV) included head trauma (8.5–27%), Ménière's disease (0.5–30%), vestibular neuritis (0.8–20%), and idiopathic sensorineural hearing loss (0.2–5%) [5–13].

The mechanical detachment of otoconia through *head trauma* is the most commonly associated condition, with the reported incidence of head trauma among BPPV patients ranging 8.5–27% [6–8, 12–14]. The nature and severity of the traumas causing trauma-BPPV are diverse, ranging from minor head injuries to more severe head and neck trauma with brief loss of consciousness. To reinforce the etiological relationship between head trauma and BPPV, we may note that the incidence of BPPV in a study of 150 consecutive severe head trauma patients has been reported to be significantly higher than in the general population (6.6%) [15]. Following the reported high incidence rates, secondary BPPV should be suspected in any case of head trauma accompanied with positional vertigo, and a Dix-Hallpike examination should be included in the diagnostic protocol of these patients, in some cases, despite the consequent patient discomfort.

The incidence of *Ménière's disease* (MD) among BPPV patients has been reported within the wide range of 0.5–30% [5–13]. Vice versa, based on a study of 500 patients with MD, it is estimated that approximately 65 to 70% of patients will experience BPPV between attacks of the disease [16]. Another interesting observation is that a significant percentage of MD patients (9/162 or 5.5%), mostly females, seem to develop intractable BPPV [17]. Therefore, the examination of MD patients should also involve the application of a Dix-Hallpike test for the exclusion of secondary BPPV. The importance of such a missed diagnosis lies, obviously, in the different therapeutical approaches, the immediacy of patient relief that may follow an appropriate repositioning manoeuvre, and the sustained efficacy of a long medical treatment for MD. 

The underlying pathophysiological mechanism seems to regard an endolymphatic hydrops-induced destruction of the maculae of the utricle and saccule either through vascular compromise or through direct distortion of its surface, resulting in detachment of otoliths into the endolymph. Incidence rate increases as the MD course is prolonged [18]. This may be explained by the hypothesis that periodic hydropic distension, as seen in the natural course of MD, may enhance detachment of otoliths through macular fibrosis [17]. Temporal bone studies have verified the existence of free-floating deposits in at least one semicircular canal of subjects with BPPV and MD, as well as significant differences in the incidence of cupular and free-floating deposits in the posterior and lateral semicircular canals between subjects with MD and healthy controls. The findings have been associated with the duration of disease rather than with aging [19]. Therefore, an exclusion of secondary BPPV should be incorporated in the clinical examination of patients with MD, especially those with a long history of the disease. 

The incidence of *vestibular neuritis* among BPPV patients has been reported within the wide range of 0.8–24.1% [9–11]. Vice versa, in patients with vestibular neuritis the incidence of BPPV appears to be more frequent (9.8–20%) than in the general population [20–22]. These percentages seem to justify the application of a Dix-Hallpike examination to patients with vestibular neuritis, as well as the performance of a detailed clinical and laboratory neurotological testing in patients with BPPV. In fact, the application of nystagmography in idiopathic BPPV patients has been reported to reveal ipsilateral canal paresis at a percentage of 13–47% [7, 14, 23]. Furthermore, the percentage of abnormal vestibular-evoked myogenic potentials (VEMPs) in BPPV patients has been reported to be statistically higher than in control ears (*P* < 0.005) [24]. Although these findings have been hypothesized to correspond to a more extensive inner ear lesion, their diagnostic and/or prognostic value remains unclear.

The pathogenetic mechanism underlying secondary BPPV in patients with vestibular neuritis seems to derive from the distribution of the vestibular nerve in the inner ear. The superior vestibular nerve innervates the cristae of the anterior and lateral SCCs and the macula of the utricle. A lesion of the lateral semicircular canal and the superior vestibular nerve is associated with abnormal nystagmographic findings. The typical superior vestibular nerve lesion sparing the inferior division of the nerve seems to be the main pathogenetic mechanism underlying BPPV in vestibular neuritis [25]. Damage to the utricle may detach the otoconia. More extended utricle damage is possibly expected to be more likely to induce the detachment of otoconia. However, the prognostic role of BPPV in patients with vestibular neuritis has not been investigated yet. After otoconia is detached from the utricle, it could enter the posterior SCC duct. The clinical signs and symptoms of posterior SCC BPPV will be presented, because this SCC is innervated from the inferior vestibular nerve. Damage to the superior vestibular nerve innervating the anterior and lateral SCCs may abolish the vestibuloocular reflex pathway from these SCCs. Therefore, posterior SCC-BPPV nystagmus is, as expected, the typical finding in BPPV patients with vestibular neuritis. This implies that at least some function in the inferior vestibular nerve remains, as it is also supported by the preserved vestibular-evoked myogenic potentials in postneurolabyrinthitis patients [26]. These potentials are most likely of saccular origin and both the macula of the saccule and the crista of the posterior canal are innervated by the inferior vestibular nerve.

*Postsurgical* BPPV seems to be another underdiagnosed entity. Surgeries involving drilling and especially maxillofacial and dental surgery including placement of dental implants [27, 28] and cochlear implantation [29, 30], have been associated with secondary BPPV. Incidence has been reported at 3% and 0–28%, respectively [28–32]. The incidence of secondary BPPV in otosclerotic patients ranges between 6.3 and 8.5%, it is developed between the 5th and 21st days after surgery and attributed to utricular trauma [33, 34]. Surgeons should probably not omit the exclusion of BPPV in patients complaining of Postsurgical dizziness by the use of a simple clinical examination, before subjecting them to imaging examinations and before a final diagnosis involving other Postsurgical complications is reached.

According to the hypothesized mechanism, drilling might detach utricular otoconia mechanically, in a similar way as head trauma does. The hypothesized mechanism underlying secondary BPPV after cochlear implantation presents some additional interesting aspects. Regarding this type of Postsurgical BPPV, a considerably heterogeneous and long time interval has been reported between surgery and initiation of BPPV symptoms (28–165 days in the study of Viccaro et al. and 1–880 days in the study of Limb et al.) [29, 30]. For the patients with delayed development of BPPV, direct falling of bone dust particles into the cochlea during cochleostomy, as well as dislodging of otoliths through, electrical stimulation, has been hypothesized to account for BPPV [29]. After falling into the cochlea, bone dust particles might, through a microrupture of the basilar membrane, travel into the endolymphatic compartment of the scala media and into the lumen of the posterior semicircular canal, thereby producing canalolithiasis and subsequent delayed-onset BPPV [30]. The hypothesis of the dislodging of otoconia because of electrical stimulation seems to be less possible, when taken into consideration that approximately 1/3 of these patients have been reported to experience symptoms before implant activation [27].

Although in most studies there is no distinction between sudden deafness and *sudden sensorineural hearing loss* (SSNHL), or whether the hearing loss was the sole symptom or part of a coexisting pathology, there seems to be an association between idiopathic sudden sensorineural hearing loss and BPPV. This pathology is encountered in 0.2–5% of BPPV patients. Vice versa, the diagnosis of BPPV has been reported for 12.7% of patients with SSNHL [35]. Therefore, patients with idiopathic sudden hearing loss and dizziness should be subjected to clinical examination for the diagnosis of BPPV, even though typical BPPV symptoms may not be described by the patient [3, 4]. Regarding the underlying pathogenetic mechanism, it is logical to hypothesise that otoconia is in these cases detached due to vascular compromise or viral lesions of the macula; however, the underlying mechanism remains actually unknown. 

The incidence of BPPV is also known to be higher in patients who suffer from *migraine*. Lempert et al. [36] found that the prevalence of migraine in patients with BPPV was twice as high as that in age- and sex-matched controls. The relationship between migraine and BPPV is poorly understood. It has been suggested that migraine causes vasospasm of the labyrinthine arteries, hence inducing local ischemia which facilitates otoconia detachment from the utricular macula [37]. 

Possibly through the same mechanism of vascular depletion of the inner ear, BPPV has been reported to occur in association with giant-cell arteritis, diabetes, osteopenia/osteoporosis and hyperuricemia [38–41]. As far as osteopenia/osteoporosis is concerned, disturbed internal structure of the otoconia or their interconnection and attachment to the gelatinous matrix and reduced capacity to dissolve the dislodged otoconia owing to increased concentration of free calcium in the endolymph have also been proposed as possible underlying mechanisms [41, 42].

### 4.2. Clinical Characteristics

BPPV secondary to mild *head trauma* has been reported to affect younger populations with more even age and gender distribution in comparison to the idiopathic form. An important difference noted by several authors is the higher incidence of bilaterality [12, 13, 43]. Most bilateral cases in both idiopathic and secondary BPPV groups seem to apply to the PSC. Some authors report no differences in the semicircular canals involved, while others note a consistently higher prevalence of the posterior (PSC) than the horizontal semicircular canal BPPV (HSC) in both groups [10, 12, 43]. Association with chronic dizziness has been reported to be similar in the two groups [43].

Bilateral involvement is also a significant characteristic of BPPV secondary to *Ménière's disease.* In the 41 patients with unilateral Ménière's disease reported by Gross et al. [17], 18 had bilateral BPPV, 16 had BPPV of the same ear, and 7 had only contralateral BPPV. The horizontal semicircular canal has been reported as the most commonly affected. Onset is usually noted within one week following an attack in the majority of patients (60%), whereas simultaneous onset is uncommon (10%) [10]. This clinical characteristic possibly implies the need for more than one diagnostic session, before BPPV is safely excluded for a MD patient. Female predominance in secondary BPPV seems to follow the current epidemiology of Ménière's disease [17, 18]. There is a literature discrepancy regarding the most commonly affected canal. The posterior as well as the lateral semicircular canal have both been reported as the most frequently involved by different authors [10, 18].

BPPV secondary to *vestibular neuritis* is expected on average as late as 18 days after the onset of the primary disease [10]. The late emergence of BPPV after vestibular neuritis may highlight the necessity for the repentance of the Dix-Hallpike examination at the follow-up sessions, especially in patients who present a slow recovery. BPPV seems to be in these cases a negative prognostic factor, since it has predominantly been diagnosed in patients who did not fully recover from the disease [20]. As it has been analysed in terms of the possible underlying pathogenetic mechanism, BPPV seems to consistently affect the posterior canal of the ipsilateral ear. 

On the contrary, more than half of the patients with secondary BPPV due to *idiopathic sudden sensorineural hearing loss* develop it relatively early, within 24 hours after the onset of deafness [35]. Information on the most commonly affected SCC(s) is not clearly stated in the relevant reports. 

Finally, *postsurgical* BPPV secondary to middle ear surgery, cochlear implantation, dental and maxillofacial surgery also affects predominantly the posterior semicircular canal possibly because the posterior SCC is situated lower than the vestibule in the supine position [27, 29, 30]. The mean onset time for BPPV after maxillofacial and dental surgery has been reported at 4.1 days after the exclusion of patients who developed BPPV seven days or later after surgery [27]. Regarding cochlear implantation and due to the specific, other than drilling, pathogenetic mechanisms that have implicated in the development of this type of secondary BPPV, authors have adopted less strict time interval criteria, by reporting on patients who developed BPPV up to 165 or 880 days after surgery [29, 30]. Bilateral involvement in these surgeries seems to be rare, in contrast to what might have been expected through the pathogenetic mechanism of the transmission of mechanical energy through the bones and perilymphatic fluids toward the maculi [29, 44]. Patient age, implant side, device type, and aetiology of hearing loss were randomly distributed with respect to likelihood of postoperative BPPV [30]. Interestingly, no postcochlear implantation BPPV cases have been reported even in large pediatric populations [45].

### 4.3. Management

Patients with idiopathic BPPV tend to present significantly higher rates of symptoms' resolution with canalith repositioning procedures (CRP) than those with secondary BPPV due to head trauma, vestibular neuritis or Ménière's disease [13, 21, 46–48]. The mean durations of treatment until complete resolution of signs and symptoms have been reported at 2.28 for idiopathic BPPV and at 4.87 days for secondary BPPV. Such differences seem to apply also among the various types of secondary BPPV. In a retrospective study of 69 patients with secondary BPPV, the mean duration of treatment has been reported to be 6.28 days for idiopathic sudden sensorineural hearing loss with BPPV, 5.07 days for BPPV with vestibular neuritis and 2.28 days for BPPV with Ménière's disease [10]. Significant differences were noted between patients with posttraumatic BPPV and patients with idiopathic BPPV regarding both complete resolution rates after a single CRP and recurrent attacks during the 6- to 42-month followup [13]. In cases, however, where a single CRP was not enough to achieve complete resolution of symptoms and signs, the number of multiple CRPs required does not seem to reach statistical significance between the secondary and idiopathic BPPV groups [13, 46]. The aforementioned differences in the management and prognosis of idiopathic and secondary BPPV may lead to the hypothesis that they may result from quantitatively or qualitatively different lesions [47]. Moreover, the diverse clinical courses in the various subtypes of secondary BPPV may be explained by the different pathophysiologies associated with variant inner ear diseases [10]. The worse prognosis of BPPV with unilateral vestibulopathy has not been verified by other authors who, in a small group of 35 patients, reported that acute vestibular neuritis patients seem to have a tendency for a better outcome than secondary BPPV patients with any other etiology [46]. The same authors have reported that resolution rates of paroxysmal positional nystagmus after CRP seem to be similar in idiopathic and secondary BPPV patients. Despite these contradicting comparisons, the authors admit that secondary BPPV is still more difficult to treat than idiopathic cases because in a considerable number (42%) of patients with secondary BPPV, persistence of positional vertigo after CRP is noted after the disappearance of nystagmus on the Dix-Hallpike manoeuvre [46]. 

Detailed neurotological studies of patients with secondary BPPV have reached the hypothesis that an additional vestibular lesion, also causing vertigo with positional triggers, may coexist with BPPV and preserve the symptoms of vertigo in some patients [46]. BPPV patients have been reported to reveal ipsilateral canal paresis at a percentage of 13–47% [7, 14, 23]. In as much as one-third of these patients, especially patients with head trauma and vestibular neuritis, the side of the paresis has been reported to be the side opposite to the ear treated for BPPV. Although the incidence of additional vestibular pathology in the contralateral side may indeed be just an incidental unrelated finding, a contrecoup lesion in the cases of head injury cannot be excluded [46]. Directional preponderance in the absence of canal paresis has been found in 22% of secondary BPPV patients, and in the absence of central vestibular abnormalities, this may also be considered as a sign of peripheral vestibular dysfunction. Therefore, the difficult management of secondary BPPV may in many cases be attributed to additional inner ear lesions. At least patients who remain symptomatic after CRP should be subjected to a more comprehensive testing of the SCCs and otolithic system. Patients with evidence of concomitant vestibular pathology would be expected to require further vestibular rehabilitation in the form of systematic or customized exercises. 

Regarding the recurrence rates of BPPV secondary to *head trauma,* there is no agreement in the literature [12, 13, 49]. Some authors report that secondary BPPV has a greater tendency to recur [12, 13], while others fail to find any differences in recurrence after repositioning manoeuvres or associated chronic dizziness [43, 49]. 

Recurrence has been consistently reported to be significantly more common in BPPV secondary to *Ménière's disease* than in idiopathic cases [18, 50]. Endolymphatic hydrops seems to be associated with higher BPPV recurrence rates [50, 51]. The higher recurrence rates may be explained by the periodic hydropic distension, which is seen in the natural course of Ménière's disease and may result in repeated release of otoconia [17]. The difficulties in the treatment of BPPV in these patients may also be attributed to the repeated hydropic distension. This may reduce the elasticity of the membranous labyrinth and result in partial collapse or adhesions of the semicircular canal membranous labyrinth which therefore exhibits partial obstruction(s) [18]. A dilated saccule, as well as adhesion of otoliths to the membranous labyrinth has also been postulated as a possible mechanism of partial obstruction [17, 52, 53]. In all the above mentioned hypotheses, partial obstruction can persist independently of Ménière's disease recurrences. With partial obstruction, canalith repositioning is impeded, although still feasible, and this may provide some explanation for the persistent BPPV and the lower responses of these patients to canalith repositioning manoeuvres [17]. There is no data neither on the possible prognostic role of BPPV in the course of MD nor on the effect of BPPV on the rehabilitation of these patients.

Although several studies have highlighted the negative prognostic value of vertigo in *idiopathic sudden sensorineural hearing loss,* the majority of cases do not regard BPPV [54, 55]. The unfavourable prognosis of patients with idiopathic sudden sensorineural hearing loss and BPPV reported by Lee and Ban [35] may need to be verified by additional studies. Such studies on the possible prognostic role of BPPV in the course of vestibular neuritis and on the effect of BPPV on the rehabilitation of these patients are also currently lacking.

Information on the management of *postsurgical* BPPV is also scarce. Most authors note that patients were successfully treated with CRP, without further commenting on the number of repositioning manoeuvres needed to resolve BPPV symptoms. In a cohort of 8 patients after cochlear implantation, Viccaro et al. [29] have reported the case of one patient with persistent BPPV that did not respond to repositioning manoeuvres and continued to have symptoms when turning the head toward the implanted side. BPPV does not seem to affect cochlear implant performance [30].

## 5. Conclusions

Secondary BPPV seems to be an underdiagnosed entity, especially among patients with known causes of vertigo such as Ménière's disease, vestibular neuritis, idiopathic sudden sensorineural hearing loss, and postsurgical patients. A higher suspicion index and the incorporation of the Dix-Hallpike test in the examination battery of all patients with vertigo, regardless of a known primary inner ear disease, may lead to the diagnosis of underlying secondary BPPV and offer those patients an optimal and efficient treatment. Reversely, BPPV seems to be associated with inner ear disease in more cases than it has been generally believed. In many patients, the initial finding of BPPV is assumed to be the final diagnosis and other neurootological tests are neglected. It is however recommended to complete the neurootological examination even if BPPV has been diagnosed with the clinical provocative tests. Especially in BPPV patients with persisting symptoms, a comprehensive audiological and neurotological evaluation should probably be performed in order to recognise any associated inner ear pathology. 

Unfortunately, the clinical differences between idiopathic and secondary BPPV demonstrated by large studies are indefinite and of limited clinical value. Most studies agree that secondary BPPV is more difficult to treat than idiopathic and patients require longer time intervals before becoming free from clinical symptoms. The clinical manifestation of an additional inner ear lesion may be a possible explanation. Therefore, a detailed medical history, and clinical, laboratory, and follow-up examination seem to be of outmost importance for the diagnosis and successful management of secondary BPPV. Further clinical studies are needed in order to investigate the possible prognostic role of secondary BPPV in various inner ear diseases as well as any differences in the time course and efficiency of rehabilitation in these patients.
